# Personal

**Published:** 1906-07

**Authors:** 


### PERSONAL.

DR. JOHN PARMENTER, professor of clinical surgery in the University of Buffalo, was presented with a loving cup by the graduates of the medical department on commencement day, June 1, 1906. The presentation was made by Dr. Luther A. Thomas on behalf of the graduated class, who stated the cup was a token of their appreciation of Dr. Parmenter's ability as an instructor.

---

DR. EDWARD J. ILL, of Newark, a Fellow of the American Association of Obstetricians and Gynecologists, was elected vice-president of the Medical Society of the State of New Jersey at its One Hundred Fortieth annual meeting held at Atlantic City, June 19-21, 1906.

---

DR. JANE WALL CARROLL, a graduate of the medical department of the University of Buffalo, 1891, took the additional degree of bachelor of laws at the recent annual commencement held June 1, 1906.

---

PROFESSOR DR. ALFRED DUHRSSEN, of Berlin, was the guest of Mr. Edward H. Butler, editor of the Buffalo News, at dinner at the country club Saturday evening, June 2, 1906. Professor Duhrssen was on his way east from Cleveland to attend the American Medical Association meeting at Boston.

Dr. Julius Pohlman, of Buffalo, accompanied by his wife and children, sailed from Montreal, June 26, 1906, for Scotland. They expect to be absent about a month.

---

Dr. B. P. Hoyer, of Buffalo, has removed his office and residence from 220 North Division Street to 46 Allen street.

---

Dr. James E. King, of Buffalo, announces the removal of his office and residence to 1248 Main street. Hours: 2 to 4 and by appointment; Sunday, by appointment only. Telephones: Bell, Bryant 1463-R; Frontier, 24742.

---

Dr. Grover W. Wende, of Buffalo, was elected secretary and treasurer of the American Dermatological Association at its annual meeting held at Cleveland, May 30 - June 1, 1906.

---

Dr. and Mrs. A. Vander Veer and Dr. James N. Vander Veer, of Albany, paid a visit to Buffalo recently, in company with their guests Mr. and Mrs. Charles A. Ballance, of London, and Mr. H. A. Ballance, of Norwich, England. After visiting Niagara Falls they proceeded to Cleveland to attend the meeting of the American Surgical Association, of which Dr. A. Vander Veer was president.

---

Dr. and Mrs. George Ben Johnston, of Richmond, visited Buffalo and Niagara Falls, June 2, 1906, on their way from Cleveland to Boston, whither they were going to attend the meeting of the American Medical Association.

---

Dr. Denslow Lewis, of Chicago, spent a few days in Buffalo recently, the guest of Dr. Ernest Wende. Dr. Lewis addressed the Alumni Association, University of Buffalo, during commencement week, his subject being, "The social evil." He went from Buffalo to Boston to preside over the Section of Hygiene and Sanitary Science of the American Medical Association.

---

Dr. M. Elizabeth Schugens, of Buffalo, was elected vice-president of the Buffalo Normal School Alumni Association at its thirtieth annual meeting held June 25, 1906. Dr. Schugens belonged to the normal school class of 1886, and took her doctorate degree from the medical department of the University of Buffalo in 1891. She is now a teacher at the Masten High School.

---

Dr. William Gaertner, of Buffalo, was elected president of the local branch of the German-American National Alliance which was organised in this city May 25, 1906. The object of the organisation is to unify and strengthen the fraternal feeling among German-Americans. It has branches in the majority of the states of the union.

---

Dr. Irving White Potter, of this city, was elected chairman of the section of obstetrics and gynecology of the Buffalo Academy of Medicine at the meeting of that section held May 22, 1906. Dr. William F. Getman was elected secretary.

---

Dr. Joseph D. Bryant, president of the Medical Society of New York, was elected president of the American Medical Association at its recent annual meeting held at Boston.

---

Dr. and Mrs. J. N. McCormack, of Bowling Green, Ky., sailed for Europe on the Majestic June 13, 1906, for rest and travel, and expect to be absent about three months.

---

Dr. William H. Wathen, of Louisville, was chosen to deliver the oration on surgery at the next annual meeting of the American Medical Association.

